# Craniopharyngioma and Metabolic Syndrome: A 5-Year Follow-Up Single-Center Experience

**DOI:** 10.3389/fneur.2022.783737

**Published:** 2022-03-09

**Authors:** Elisabetta Scarano, Domenico Solari, Enrico Riccio, Rossana Arianna, Teresa Somma, Luigi Maria Cavallo, Fiammetta Romano, Annamaria Colao, Carolina Di Somma

**Affiliations:** ^1^Endocrinology Unit, Department of Clinical Medicine and Surgery, University of Naples Federico II, Naples, Italy; ^2^Division of Neurosurgery, Department of Neurosciences, Reproductive and Odontostomatological Sciences, University of Naples Federico II, Naples, Italy; ^3^Cattedra Unesco “Educazione alla salute e allo sviluppo sostenibile”, University of Naples Federico II, Naples, Italy

**Keywords:** craniopharyngioma, obesity, metabolism, cardiovascular, hypothalamic

## Abstract

Patients with craniopharyngioma often have comorbidities, such as obesity and hypopituitarism. These two conditions affect each other and worsen the quality of life of patients, which lead to a higher risk of morbidity and mortality. In addition, abdominal obesity, measured as waist circumference (WC), is together with other parameters [arterial hypertension, hyperglycemia, hypertriglyceridemia, and reduced levels of high-density lipoprotein (HDL) cholesterol], one of the components of metabolic syndrome (MS). Each one of these morbidities occurs in patients with craniopharyngioma more frequently than in the remaining population. On these bases, we evaluated metabolic parameters in patients with craniopharyngioma at the time of diagnosis and after a 5-year follow-up, which compares these data with those of age-, gender-, WC-, and body mass index (BMI)-matched controls. In addition, we evaluated the prevalence of MS according to IDF criteria (MS-IDF) and the prevalence of MS according to ATP III (MS-ATPIII) criteria in patients and controls at baseline and after 5 years. We recruited 20 patients with craniopharyngioma (age 38.5 ± 15 years, 10 M) and 20 age-, gender-, WC- and BMI-matched controls (age 34.16 ± 13.19 years, 10 M). In all patients and controls, we evaluated the following: anthropometric features [height, weight, BMI, WC, hip circumference (HC) and waist-to-hip ratio (WHR)], systolic blood pressure (SBP) and diastolic blood pressure (DBP), lipid profile [total cholesterol (TC), HDL, low-density lipoprotein (LDL) cholesterol, triglycerides (TG)], and blood glucose at baseline and after 5 years. The prevalence of MS, according to IDF and ATPIII criteria, was calculated in the two groups at baseline and after 5 years. According to our results, at baseline, patients with craniopharyngioma had a worse metabolic profile than controls and a higher prevalence of MS. Besides, at a 5-year follow-up, patients still had impaired metabolic characteristics and more frequent MS (according to IDF and ATPIII criteria) when compared to controls. These data confirm that MS in patients with craniopharyngioma is unresponsive to life-changing interventions and to a common pharmacological approach. Other factors may be involved in the evolution of these conditions; so, further studies are needed to establish the correct management of these patients.

## Introduction

Craniopharyngiomas represent 1–15% of all primary intracranial neoplasms (1–3) and 5.6–15% of intracranial tumors of children (2). Although it is the most common lesion involving the hypothalamic–pituitary area in children, about half of cases are diagnosed in adults (1, 3, 4), with a peak incidence around 65–74 years. The symptoms associated with the presence of craniopharyngiomas are due to the development of a space-occupying mass in a non-expandable region and are characterized by headache, visual changes, hydrocephalus, pituitary deficits, and hypothalamic disease (diabetes insipidus, obesity, dysphoria, numbness, temporal-spatial disorientation and alterations in the sense of hunger or thirst, sleep–wake rhythm, and thermoregulation) (5–12). In particular, the hypothalamic obesity associated with this condition is believed to be due to an alteration of both the signals that reach the hypothalamus from the periphery, mainly mediated by leptin, an anorectic action protein that induces a reduction in the sense of hunger and an increase of energy expenditure, and the signal that goes from the hypothalamus to the periphery, with an increase in vagal tone, insulin secretion, and adipogenesis (13–15).

Obesity is associated with severe metabolic and psychological consequences, inducing comorbidities, reduced quality of life, and life expectancy (13, 14). Furthermore, abdominal obesity, measured as waist circumference (WC), is, with other parameters such as arterial hypertension, hyperglycemia, hypertriglyceridemia, and reduced high-density lipoprotein (HDL) cholesterol levels, one of the components of the metabolic syndrome (MS), the elements of which occur, in patients with craniopharyngioma, more frequently than in the remaining population (16, 17).

On these bases, this study aimed to evaluate metabolic status in patients with craniopharyngioma at the time of diagnosis and after a 5-year follow-up, which compares these data with controls.

## Materials and Methods

The study was approved by the local ethics committee and complied with the Declaration of Helsinki, in line with the Guidelines for Good Clinical Practice. All patients provided written informed consent before entering the study, with respect to study participation, and confidentiality statement of data collection according to the Italian privacy policy.

### Patients and Controls

We consequently recruited 20 patients with craniopharyngioma (age 38.5 ± 15 years, 10 M) followed at the outpatient clinic of the Department of Clinical Medicine and Surgery, Section of Endocrinology, the University of Naples“Federico II,” Italy, from 2012 to 2018. All patients underwent surgical treatment for craniopharyngioma. In five patients, a subsequent radiotherapy treatment was also necessary (Table 1). Multiple pituitary deficits developed following these treatments, and therefore, where necessary, patients underwent replacement therapy with glucocorticoids, rhGH, thyroid hormones, testosterone, estrogen and progestogen, and desmopressin. Table 2 shows pituitary deficits found at baseline and after 5-year follow-up (Table 2). We also recruited 20 controls (age 34.16 ± 13.19 years, 10 M) followed at the Department of Clinical and Surgical Medicine, Section of Endocrinology, the University of Naples“Federico II” between 2012 and 2018 for outpatient checkups, in which no endocrinological disease was found.

**Table 1:** Patients' treatments.

**Treatment**	**No. of Patients**
Surgery	15
Surgery + Radiotherapy	5
Total	20

**Table 2:** Number of pituitary deficiencies in the study population at baseline and after 5-year follow-up.

**No. of deficiencies**	**Baseline**	**5 years**
Isolated deficiency	0	0
1 additional deficiency	1	1
2 additional deficiencies	8	3
>3 additional deficiencies/3+DI	11	16

*DI, Diabetes insipidus*.

The prevalence of MS, according to IDF and ATPIII criteria, was calculated in the two groups at baseline and after 5 years. In both groups, moreover, there were patients undergoing therapy for hyperlipidemia, impaired glucose tolerance, and hypertension. These criteria were considered for the diagnosis of MS.

The characteristics of patients with craniopharyngioma (group 1) and controls (group 2) at the time of recruitment for the study and after 5-year follow-up are shown in Tables 3, 4. The two groups were comparable in age, gender, WC, and body mass index (BMI) at baseline (Table 5).

**Table 3:** Clinical features of patients (group 1) at baseline and after 5-year follow-up.

**Parameters**	**Baseline**	**5 years**	***P***
BMI (kg/m^2^)	30.92 ± 8.19	32.74 ± 9.41	NS
WC (cm)	100.15 ± 18.63	101.95 ± 19.9	NS
HC (cm)	106.90 ± 13.39	110.35 ± 13.88	NS
WHR	0.91 ± 0.17	0.92 ± 0.14	NS
SBP (mmHg)	120.9 ± 12.58	120.8 ± 14.85	NS
DBP (mmHg)	73.35 ± 8.1	78.0 ± 11.4	NS
TC (mg/dL)	222.8 ± 39.56	203.0 ± 39.4	NS
LDL (mg/dL)	150.23 ± 39.38	133.63 ± 35.02	NS
HDL (mg/dL)	41.9 ± 10.18	40.5 ± 11.8	NS
TG (mg/dL)	152.3 ± 48.03	147.5 ± 51.06	NS
Fasting glucose (mg/dL)	98.3 ± 15.2	97.03 ± 12.87	NS

*BMI, body mass index; WC, waist circumference; HC, hip circumference; WHR, waist-to-hip ratio, SBP, systolic blood pressure; DBP, diastolic blood pressure; TC, total cholesterol; LDL, low-density lipoprotein cholesterol; HDL, high-density lipoprotein cholesterol; TG, triglycerides*.

**Table 4:** Clinical features of controls (group 2) at baseline and after 5-year follow-up.

**Parameters**	**Baseline**	**5 years**	***P***
BMI (kg/m^2^)	29.17 ± 6.17	29.08 ± 6.18	NS
WC (cm)	99.22 ± 17.55	98.8 ± 17.16	NS
HC (cm)	106.87 ± 12.75	105.98 ± 11.73	NS
WHR	0.92 ± 0.08	0.93 ± 0.09	NS
SBP (mmHg)	114.3 ± 4.3	100.1 ± 13.3	0.000
DBP (mmHg)	72.8 ± 8.61	68.31 ± 13.42	NS
TC (mg/dL)	183.56 ± 41.59	180.4 ± 26.6	NS
LDL (mg/dL)	105.76 ± 38.5	101.6 ± 32.12	NS
HDL (mg/dL)	52.4 ± 11.51	54.62 ± 11.61	NS
TG (mg/dL)	128.04 ± 33.86	123.12 ± 38.41	NS
Fasting glucose (mg/dL)	92.33 ± 14.84	91.74 ± 10.32	NS

*BMI, body mass index; WC, waist circumference; HC, hip circumference; WHR, waist-to-hip ratio, SBP, systolic blood pressure; DBP, diastolic blood pressure; TC, total cholesterol; LDL, low-density lipoprotein cholesterol; HDL, high-density lipoprotein cholesterol; TG, triglycerides*.

**Table 5:** Comparison between patients (group 1) and controls (group 2) at baseline and after 5-year follow-up.

	**Baseline**		**5 years**	
**Parameters**		***P* Group** **1 vs. 2**		***P* Group** **1 vs. 2**
BMI		NS		NS
WC		NS		NS
HC		NS		NS
WHR		NS		NS
SBP		0.032^a^		0.000^a^
DBP		NS		0.018^a^
TC		0.004^a^		0.040^a^
LDL		0.000^a^		0.005^a^
HDL		0.004^b^		0.000^b^
TG		NS		NS
Fasting glucose		NS		NS

*a: 1 > 2; b 2>1*.

*BMI, body mass index; WC, waist circumference; HC, hip circumference; WHR, waist-to-hip ratio, SBP, systolic blood pressure; DBP, diastolic blood pressure; TC, total cholesterol; LDL, low-density lipoprotein cholesterol; HDL, high-density lipoprotein cholesterol; TG, triglycerides. NB, data of parameters of each group are shown in Tables 3, 4*.

### Parameters

All parameters were evaluated at baseline and after 3 months during the first year of follow-up and every 6 months during the following 5 year follow-up.

In all patients and controls, we evaluated anthropometric features [height, weight, BMI, WC, hip circumference (HC), and waist-to-hip ratio (WHR), systolic blood pressure (SBP), and diastolic blood pressure (DBP)] at baseline and after 5-year follow-up.

Body weight was measured in the morning to the nearest 0.1 kg, and body height was measured to the nearest 0.01 m when the subject was wearing light indoor clothes without shoes. BMI was calculated as body weight (in kilograms) divided by squared height (in squared meters). WC was measured with a soft tape, midway between the lowest rib margin and the iliac crest, in the standing position. HC was measured over the widest part of the gluteal region, and then, WHR was calculated.

Blood pressure was measured at the right arm with the patient in the sitting position. The average of three measurements using a mercury sphygmomanometer was calculated. Hypertension was diagnosed when SBP exceeded 140 mmHg and DBP exceeded 90 mmHg.

We also evaluated in both groups: lipid profile [total cholesterol (TC), HDL, LDL (low-density lipoprotein) cholesterol, triglycerides (TG)], and blood glucose at baseline and after 5 years. Fasting venous blood sampling was performed between 8 and 12 a.m. The blood samples were immediately centrifuged, and the sera were stored at a temperature of −80°C until they were analyzed. Serum levels of TC, HDL, and triglycerides were determined by an enzymatic method on fasting serum. LDL was calculated according to Friedewald's formula adjusted to SI units (18). Serum LDL-C was excluded in patients with serum triglycerides >400 mg/dl. Blood glucose was measured by fasting on serum or plasma samples.

The presence of MS was evaluated in the two groups at baseline and after 5 years according to both the IDF and the ATPIII criteria. Thus, using the IDF criteria, the subject with abdominal obesity, defined as a WC >94 cm for men and WC >80 cm for women, was considered to have MS, with at least two of the following factors: high values of TG value: >150 mg/dL or specific treatment for such dyslipidemia, reduced HDL-C values: <40 mg/dL in men and <50 mg/dL in women or hypercholesterolemia, high BP >130/85 mm/Hg or hypertension treatment, high fasting blood glucose: >100 mg/dL or diagnosis of type 2 diabetes mellitus (19, 20). Using the ATPIII criteria, the subject was considered to have MS if they had three of the following factors: abdominal obesity, defined as a WC >102 cm for men and WC >88 cm for women, high values of TG value: >150 mg/dL or specific treatment for such dyslipidemia, reduced HDL values: <40 mg/dL in men and <50 mg/dL in women or hypercholesterolemia, high BP > 130/85 mm/Hg or hypertension treatment, high fasting blood glucose: >100 mg/dL or diagnosis of type 2 diabetes mellitus (21). Hypopituitarism was diagnosed based on the clinical manifestations, the baseline assessment of pituitary function, and the stimulus test, according to the Clinical Practice Guidelines of the Endocrine Society (22). Peripheral venous blood samples were taken in the morning between 8 and 10 a.m., after fasting for at least 8 h and conserving at −80°C until processing. Hormonal profile evaluation [growth hormone (GH), insulin-like growth factor 1 (IGF-1), thyroid-stimulating hormone (TSH), follicle-stimulating hormone (FSH), luteinizing hormone (LH), adrenocorticotropic hormone (ACTH), cortisol, and prolactin] was performed by chemiluminescence immunoassay (CLIA). We performed biochemical assays [GH, IGF-I, TSH, free thyroxine (FT4), free triiodothyronine (FT3), FSH, LH, estradiol, testosterone, ACTH, cortisol, and prolactin] and thyroid and abdomen ultrasound in controls to exclude endocrinological diseases.

### Statistical Analysis

Data were reported as mean ± SD or as percentages. For all variables, within-group differences were calculated using a repeated-measures ANOVA, followed by a *post hoc* analysis performed using Bonferroni or Student–Newman–Keuls tests where applicable. The Student's *t*-test was used for intergroup–intragroup comparison. Then, the prevalence of MS according to both IDF and ATPIII criteria was calculated for the evaluation of intergroup–intragroup differences, with Fisher's exact test; significance was set at 5%. Data were analyzed using the SPSS Software (PASW version 21.0, SPSS Inc., Chicago, IL, USA) and MedCalc® package (version 12.3.0 1993-2012 MedCalc Software bvba-MedCalc Software, Mariakerke, Belgium).

## Results

### Body Composition and Blood Pressure

There was no statistical difference in the patient group and in the control group at baseline and after 5 years in body composition (weight, BMI, WC, HC, WHR) (Tables 3, 4) and between patients and controls for the same parameters at the two study time points (Table 5). Some differences were found in blood pressure. In particular, SBP was higher in patients than controls at baseline and after 5-year follow-up (*p* = 0.032 and *p* = 0.000, respectively) (Table 5). Moreover, in the control group, SBP significantly increased after 5 year follow-up (*p* = 0.000) (Table 4). At the end of the study, DBP was higher in patients than controls (*p* = 0.018) (Table 5).

### Lipid Profile and Glucose Metabolism

Total cholesterol and LDL were found higher in the patient group than the control group at baseline (TC; *p* = 0.004, LDL; *p* = 0.000) and after 5-year follow-up (TC; *p* = 0.040, DL; *p* = 0.005) (Table 5). HDL was found higher in controls than patients at the start and at the end of the study (*p* = 0.004 and *p* = 0.000, respectively) (Table 5). No differences between the two groups were found for TG and blood glucose (Table 5).

### Prevalence of MS

At baseline and after 5-year follow-up, no differences were found in the patient group (Table 6) and the control group (Table 7) regarding the prevalence of MS. At baseline, patients had a significantly higher prevalence of MS than controls according to IDF criteria and a near significantly higher prevalence according to ATPIII criteria (*p* = 0.037 and *p* = 0.058, respectively). At the end of the study, the prevalence of MS was higher in patients according to both IDF and ATPIII criteria (*p* = 0.028 and *p* = 0.028, respectively) (Table 8).

**Table 6:** The prevalence of metabolic syndrome (MS) in patients (group 1) at study entry and after 5-year follow-up.

**Patients**		**MS-IDF** ***n* (%)**	**MS-ATPIII** ***n* (%)**
Group 1	Baseline	6 (30)	7 (35)
(20 patients)	5 years	8 (40)	8 (40)
	*P*	NS	NS

*MS-IDF, metabolic syndrome according to IDF criteria; MS-ATPIII, metabolic syndrome according to ATPIII criteria*.

**Table 7:** The prevalence of MS in controls (group 2) at study entry and after 5-year follow-up.

**Patients**		**MS-IDF** ***n* (%)**	**MS-ATPIII** ***n* (%)**
Group 2	Baseline	1 (5)	2 (10)
(20 patients)	5 years	2 (10)	2 (10)
	*p*	NS	NS

*MS-IDF, metabolic syndrome according to IDF criteria; MS-ATPIII, metabolic syndrome according to ATPIII criteria*.

**Table 8:** Differences in prevalence of MS at study entry and after 5-year follow-up between patients (group 1) and controls (group 2).

	**Group**	**MS-IDF** ***n* (%)**	**MS-ATPIII** ***n* (%)**
Baseline	1	6 (30)	7 (35)
	2	1 (5)	2 (10)
	*p*	0.037	0.058
5 years	1	8 (40)	8 (40)
	2	2 (10)	2 (10)
	*P*	0.028	0.028

*Group 1, patients; group 2, controls; MS-IDF, metabolic syndrome according to IDF criteria; MS-ATPIII, metabolic syndrome according to ATPIII criteria*.

## Discussion

This is a single-center retrospective observational study on the long-term prevalence of MS in adult patients with craniopharyngioma compared with age-, sex-, WC-, and BMI-matched controls.

Up to now, the studies that have investigated the metabolic aspect of this disease have been conducted above all in pediatric patients (16, 17, 23, 24). Some studies have evaluated the prevalence of MS in pediatric patients with craniopharyngioma (16, 17, 23, 24). Srinivasan et al. (16) and Sahakitrungruang et al. (17) found that children and adolescents treated for craniopharyngioma had a higher prevalence of altered MS parameters, in particular abdominal obesity and lipid profile, compared with healthy controls of the same age, sex, BMI, and pubertal status. Moreover, these conditions were directly correlated with age: the greater the age, the greater the alteration.

Metabolic impairments that appear in patients with craniopharyngioma are probably due to the hypothalamic involvement, because of the prevalent localization of craniopharyngiomas near the hypothalamus. In fact, the extension of tumor at this level can cause an alteration in the ventromedial portion of the hypothalamus and can induce, through a wrong regulation of hunger and satiety mechanisms, severe obesity (25, 26).

In particular, some authors (13, 25) have studied the pathogenetic mechanism that leads to “hypothalamic” obesity by identifying alterations both in the afferent signal, from the periphery to the hypothalamus, and in the efferent one, from the hypothalamus to the periphery. In fact, in these patients, leptin, a protein with an anorectic action that reduces the sense of hunger and increases energy expenditure, has an impaired function that is associated with increased insulin secretion, vagal tone, and adipogenesis (13, 15, 25). Moreover, a higher preoperative BMI seems to be an important risk factor for developing postoperative hypothalamic obesity (27).

On the other hand, it is known that obesity is linked to metabolic and psychological consequences, and it is also associated with arterial hypertension, hyperglycemia, hypertriglyceridemia, and reduced levels of HDL cholesterol, which are the components of the MS (28, 29).

Some authors focused metabolic attention on the adult population (30–33), and our results are in agreement with these, which confirm a higher prevalence of MS in patients with craniopharyngioma in adulthood. In particular, the major alterations regarded the lipid profile (30, 31).

In this study, at baseline, all subjects were selected to match BMI and WC as well as gender and age.

After 5 years of follow-up, although there was a higher prevalence of MS in patients than controls, weight and BMI were not different. This could be partially justified by the fact that MS in our patients is related not much to weight or BMI but to other parameters.

In fact, in our cohort, we found higher levels of TC and LDL values in patients at the first and the late evaluation compared with controls. In addition, in our study, patients had a worse HDL profile than controls.

Furthermore, in our sample, SBP and DBP were significantly higher in patients than controls after 5-year follow-up. These conditions contribute to the finding of higher MS prevalence in this group and are in line with literature data (31).

In our patients' group, metabolic parameters did not significantly improve during follow-up, despite the disease treatment and usual medical indications for metabolic impairments. Probably, other non-classical mechanisms are involved in the metabolic impairment of patients with craniopharyngioma. In this context, a new hypothesis spreads about the alterations of circadian rhythm (34).

Our findings reveal that patients with craniopharyngioma have a higher cardiometabolic risk than the control population. In fact, cardiometabolic risk is defined as a condition favorable to the development of type 2 diabetes mellitus and cardiovascular pathologies (35, 36). Key elements are abdominal obesity, insulin resistance, and metabolic abnormalities, such as high levels of triglycerides, total and LDL cholesterol levels, and low HDL cholesterol levels (37).

This is one of the few Italian studies to consider only patients with craniopharyngioma compared to the general population in a single center for a such long follow-up period. Points of the strength of this study are the presence of a control group, the absence of them in some of the studies analyzed (30, 32, 33), and the single-center recruitment reducing the possibility of any selection bias.

Limitations of this study are the absence of data on ethnicity, lifestyle factors, and histological subtypes of craniopharyngioma. Furthermore, the body composition and the percentage of fat have not been measured with dual-energy X-ray absorptiometry (DXA), which is considered the best predictor of the risk of developing cerebrovascular and cardiovascular events (38, 39).

Another limitation is the relatively small number of samples even though we are talking about a rare disease.

In conclusion, this study demonstrated a higher prevalence of MS in patients with craniopharyngioma compared with the general population.

An alteration in the lipid profile and blood pressure was evident in patients compared with controls, but there were no significant differences with the values recorded at baseline and at 5-year follow-up, by considering the two groups separately. It remains to be established whether, in patients with craniopharyngioma, other non-classical mechanisms are involved in metabolic impairment. In particular, there is some interest in the evidence of a correlation among circadian rhythm alterations, different chronotypes, and diseases. According to this evidence, the sleep–wake regulatory system and circadian rhythm synchronization could play an additional key role to provide new indications in the management of craniopharyngioma comorbidities. Further studies, therefore, appear necessary in this regard. It may be useful, in the future, for larger-scale and longer follow-up studies to analyze the increase in the prevalence of MS and mortality in adult patients with craniopharyngioma.

## Data Availability Statement

The raw data supporting the conclusions of this article will be made available by the authors, without undue reservation.

## Ethics Statement

The studies involving human participants were reviewed and approved by Comitato Etico Università Federico II, Dipartimento di Medicina Pubblica e della Sicurezza Sociale-Sezione di Medicina Legale, Università Federico II, Naples Italy. The patients/participants provided their written informed consent to participate in this study.

## Author Contributions

ES and CD were responsible for the concept of this paper and drafted the manuscript. DS, ER, RA, TS, LC, FR, and AC provided a critical review of the paper. All authors contributed to the article and approved the submitted version.

## Conflict of Interest

The authors declare that the research was conducted in the absence of any commercial or financial relationships that could be construed as a potential conflict of interest.

## Publisher's Note

All claims expressed in this article are solely those of the authors and do not necessarily represent those of their affiliated organizations, or those of the publisher, the editors and the reviewers. Any product that may be evaluated in this article, or claim that may be made by its manufacturer, is not guaranteed or endorsed by the publisher.
